# Experimental Investigation on Pool Boiling Heat Transfer Performance Using Tungsten Oxide WO_3_ Nanomaterial-Based Water Nanofluids

**DOI:** 10.3390/ma13081922

**Published:** 2020-04-19

**Authors:** Mohammed Saad Kamel, Ferenc Lezsovits

**Affiliations:** 1 Department of Energy Engineering, Faculty of Mechanical engineering, Budapest University of Technology and Economics, 1111 Budapest, Műegyetem rkp.3, Hungary; lezsovits@energia.bme.hu; 2 Department of Mechanical Techniques, Al-Nasiriya Technical Institute, Southern Technical University, Thi-Qar 64001, Al-Nasiriya, Iraq

**Keywords:** pool boiling, nanofluids, tungsten oxide, PBHTC, experimental investigation, enhancement ratio

## Abstract

This study aims to experimentally investigate the pool boiling heat transfer coefficient behavior using tungsten oxide-based deionized water nanofluids and comparing them to deionized water as conventional fluid. The influence of different dilute volumetric concentrations (0.005%–0.05% Vol.) and applied heat fluxes were examined to see the effect of these parameters on the pool boiling heat transfer performance using nanofluids from a typical horizontal heated copper tube at atmospheric pressure conditions. Results demonstrated that the pool boiling heat transfer coefficient (PBHTC) for both deionized water and nanofluids increased with increasing the applied heat flux. The higher PBHTC enhancement ratio was 6.7% for a volume concentration of 0.01% Vol. at a low heat flux compared to the deionized water case. Moreover, the PBHTC for nanofluids was degraded compared to the deionized water case, and the maximum reduction ratio was about 15% for a volume concentration of 0.05% Vol. relative to the baseline case. The reduction in PBHTC was attributed to the deposition of tungsten oxide nanoflakes on the heating surface during the boiling process, which led to a decrease in the density of the nucleation sites.

## 1. Introduction

Boiling heat transfer is a significant heat transfer mode among other heat transfer modes due to the large heat quantity that could be removed by the latent heat of vaporization in small temperature differences. Nucleate pool boiling regime, and two-phase flow have a variety of industrial applications such as boiler tubes in power plant, evaporators in refrigeration systems, cooling of macro and microelectronic devices, and cooling of nuclear reactors, etc. [1,2,3]. Removing high heat dissipation with a miniaturization system has always been a key point for designing efficient heat exchange systems. Thus, intensifying heat transfer performance during the boiling process is essential for saving energy and keep those systems durable. During the last decades, many efforts have been made to experimentally study the pool boiling heat transfer performance with various working fluids involving pure liquids [4,5], refrigerants [6,7], and mixtures liquids [8,9,10]. Other studies have been conducted by surface heating modifications to enhance the pool boiling heat transfer performance [11,12,13,14]. One of the alternative ways to improve the thermal conductivity of the working fluid is to use nanofluids, which were introduced in 1995 by Choi and his team [15]. Nanofluids are a new class of thermo-fluids that consist of ultrafine materials (nanomaterials) suspended in conventional fluids such as water, refrigerant, oil, and any other type of liquid [16,17]. During recent years, a considerable amount of studies were reported for pool boiling heat transfer using nanofluids. Although, due to the contradictory results regarding those studies, efforts are still ongoing to thoroughly understand the mechanism behind the boiling using nanofluids. Many researchers have shown the enhancement for pool boiling heat transfer coefficient using nanofluids while other research groups reported a degradation in pool boiling heat transfer coefficient (PBHTC) using nanofluids. Several parameters were proposed in the literature that could influence the pool boiling using nanofluids performance including low stability of nanofluids due to the sedimentation and agglomeration of nanoparticles, the presence of the surfactant in order to stabilize the nanofluid suspension, surface topology of the heater, the nanomaterials morphology and the concentration, the thermophysical properties of the nanofluids, and the boiling time. Different types of nanomaterials were used to examine the pool boiling heat transfer performance such as metal nanopowders (i.e., Cu, Al, Ag), oxide metals (i.e., Al_2_O_3_, TiO_2_, CuO, ZnO, Fe_2_O_3_), carbon nanotube, and graphene. Earlier, in 1986, Yang and Maa [18] conducted a careful experimental study to clarify the behavior of pool boiling heat transfer using dispersed alumina particles with a dilute concentration between 0.1 and 0.5 wt.%. Their results showed an enhancement in pool boiling heat transfer using alumina particles.

Later, Das et al. [19] studied the effect of adding alumina nanoparticles on the behavior of the pool boiling curve and heat transfer coefficient from a narrow horizontal heated tube. Their results have shown that the mechanism of pool boiling is different from small tubes to those of typical industrial pipes due to the sliding of bubbles from the bottom part to the upper region. They also demonstrated that the pool boiling performance decreased while adding those nanoparticles. 

Kathiravan et al. [20] conducted an experimental study to examine the copper-based water nanofluid. Their results demonstrated that the pool boiling heat transfer coefficients decrease with increasing particle concentrations while increase when adding surfactant for nanofluids.

Suriyawong and Wongwises [21] studied the pool boiling performance of TiO_2_ nanoparticles-based water nanofluid at various volume fractions. They used two types of heating surfaces (copper and aluminum tubes) with different surface roughness. Their results demonstrated that the pool boiling heat transfer coefficient was enhanced for a copper tube with a concentration of 0.0001% Vol., while there was a degradation with the aluminum tube.

Cieśliński and Kaczmarczyk [22] studied the heat transfer performance experimentally during pool boiling of two types of nanofluids (Al_2_O_3_ and Cu nanoparticle-based water nanofluids). They used a horizontal smooth copper and stainless steel tubes as heating element. Nanoparticles were tested at a mass concentration of 0.01–1 wt.%. Their results showed that the concentration of nanofluids has almost no influence on the heat transfer coefficient, while the higher heat transfer coefficient was recorded for stainless steel tube compared to copper tube for the same applied heat flux. Again Cieśliński and Kaczmarczyk [23] examined the influence of operating pressure (200, 100, and 10 kPa) on two types of nanofluids during the pool boiling process. Their results revealed that independent of nanoparticle material type (i.e., Al_2_O_3_ and Cu) and concentration, an increase in operating pressure enhanced the heat transfer performance.

Kole and Dey [24] presented an experimental study on pool boiling heat transfer performance of ZnO–ethylene glycol (EG) nanofluid from a horizontal copper tube. The nucleate pool boiling heat transfer performance of ZnO–EG nanofluids with different volumetric fractions of ZnO nanoparticles was tested at atmospheric pressure from a cylindrical polished copper heating surface. The pool boiling heat transfer coefficient enhanced with ZnO nanofluid of about 22% was compared to that of EG for 1.6% Vol. concentration. Sarafraz et al. [25] experimentally studied the pool boiling heat transfer coefficient of Al_2_O_3_ based water–glycerol mixture nanofluid under atmospheric pressure. Results demonstrated that the pool boiling heat transfer coefficient increased with the presence of alumina nanofluid compared to base fluids and this improvement considerably improved with a higher volume fraction of nanoparticles.

Cieśliński and Kaczmarczyk [26] studied pool boiling of Al_2_O_3_ and Cu-based water nanofluids on rough and porous-coated horizontal tubes. Their experimental study was conducted under different pressures and nanoparticles concentrations. Their results observed that there was an enhancement for boiling performance using nanofluids on rough stainless steel tubes, and this enhancement increased with increasing system pressure. While for a porous-coated tube, there was a reduction in boiling performance with an increasing concentration of nanofluid. They concluded that the pressure system, surface modification, and concentrations of nanoparticles could affect the pool boiling performance. Sarafraz and Hormozi [27] performed a set of experiments to investigate the pool boiling heat transfer coefficient of dilute copper oxide-based water nanofluids at mass concentrations ranging from 0.1% to 0.4%). They studied the influence of a surfactant as a surface-active agent additive on the pool boiling heat transfer coefficient of nanofluids. Furthermore, other operating parameters were considered, such as heat flux and concentrations of nanofluids. Results have shown a significant reduction in the heat transfer coefficient of nanofluids compared with the base fluid without using surfactants; however, in the presence of a surfactant, it led to a higher pool boiling heat transfer coefficient compared to water. He et al. [28] experimentally investigated the pool boiling heat transfer coefficient behavior of ZnO nanoparticles based on deionized water and ethylene glycol nanofluids in a cylindrical vessel under atmospheric pressure. Their results demonstrated that increasing heat flux significantly grows the heat transfer coefficient of nanofluids. Moreover, an enhancement of critical heat flux for nanofluids was observed due to surface wettability reduction and nanoparticle coating on the heater surface.

Manetti et al.’s [29,30] experiment presented the pool boiling heat transfer coefficient of deionized water and Al_2_O_3_ nanoparticle-based water nanofluid at saturation conditions. They used different volume concentrations for nanoparticles deionized with smooth and roughness surfaces as a test section. Results observed that an increase in the heat transfer coefficient of up to 75% and 15% for the smooth and roughness surfaces, respectively, in comparison to that of water, were determined. Moreover, they examined the effect of time on pool boiling performance, and they revealed that during the 240 min of boiling there was no effect on the pool boiling heat transfer coefficient. Ciloglu [31] experimentally studied the nucleate pool boiling heat transfer of SiO_2_ nanoparticle-based water nanofluid at atmospheric pressure and saturated conditions. His results showed that the nucleate pool boiling heat transfer coefficient of the nanofluids is lower than that of deionized water, especially with high heat fluxes. This was due to the reduction in active nucleation sites and the formation of extra thermal resistance due to blocked vapor in the porous layer near the heating surface.

Karimzadehkhouei et al. [32] studied the pool boiling of two types of nanofluids (TiO_2_/water and CuO/water) on the flat copper heater at atmospheric condition. Their results have shown that for TiO_2_/water nanofluids, the pool boiling heat transfer coefficient was increased at dilute volume fraction (i.e., 15% at 0.001% wt.). In addition, the pool boiling heat transfer coefficient of CuO/water nanofluid was about (35%) compared to water as a baseline case. Shoghl et al. [33] experimentally investigated the effect of three types of nanofluids on the pool boiling heat transfer coefficient from a horizontal stainless steel tube. Their results have shown that with Al_2_O_3_ and ZnO-based water nanofluids, there was a reduction in the pool boiling heat transfer coefficient while there was an enhancement in the carbon nanotubes CNTs-based water nanofluid with the presence of a surfactant compared to the base fluid.

Norouzipour et al. [34] experimentally studied the effect of silica nanoparticle-sized based water on the pool boiling heat transfer coefficient during atmospheric pressure. The results obtained for nanofluid pool boiling on the copper surface indicate that at all the considered concentrations and nanoparticle sizes (except nanoparticles of 70 nm in size and concentration of 0.1% Vol.), the pool boiling heat transfer coefficient for the nanofluid was reduced compared to the pure water. Moreover, by increasing the diameter of silica nanoparticles from 11 to 70 nm, the pool boiling heat transfer coefficient was increased. Modi et al. [35] experimentally investigated the pool boiling performance of alumina nanofluid. Results showed that an increase in PBHTC was observed with an increase in the concentration of nanoparticles as well as for the nanoparticle-deposited surfaces. From all reported studies in literature, noticeable contradictory results regarding the pool boiling heat transfer coefficient were found during the pool boiling heat transfer using nanofluids. Therefore, more experimental investigations need to be conducted with various types and sizes of nanomaterials at different concentrations and operating conditions to thoroughly understand the behavior of nanofluids during a pool boiling heat transfer mode.

According to the author’s best knowledge, and from all reported pool boiling of nanofluids experimental studies in literature, it can be noted that there was no study to test the tungsten oxide WO_3_ nanoflakes-based deionized water nanofluid in the pool boiling process with a horizontal tube heater as a typical tube. Tungsten oxide nanopowder was considered as an attractive nanomaterial due to several merits such as good physical, optical, and chemical properties, reasonable price, being used for wastewater treatment, and solar still desalination as nanoparticles additives. However, the present research aims to experimentally investigate the pool boiling heat transfer performance for the first time using WO_3_-based water nanofluid. Different volumetric concentrations of nanoparticles (0.005%, 0.01%, and 0.05% Vol.) with a range of applied heat flux of about 15 to 130 kW/m^2^ were tested to show the influence of this new nanofluid on pool boiling heat transfer from a horizontal copper heated tube with a typical diameter (22 mm) at atmospheric pressure condition.

## 2. Experimental Methods

### 2.1. Nanofluid Formation Method

**Figure 1:**
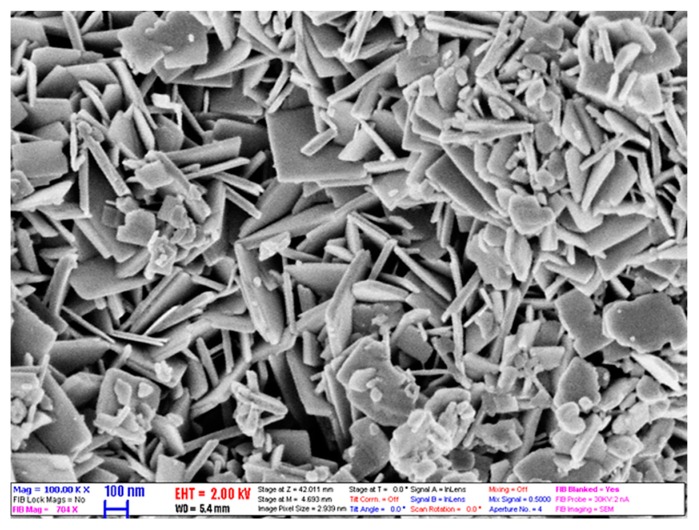
Scanning electronic microscope SEM image of tungsten oxide nanoflakes.

In this study, we used the two-step method to prepare tungsten oxide WO_3_-based water nanofluids. In this method, the dry nanopowder with a specified quantity was scaled by using an electronic balance (with an accuracy of 0.001 gram). Next, this quantity dispersed into deionized water to obtain the desired volume concentration of the nanofluids by using a conversion formula, which is used by [36,37], as shown in Equation (1).

(1) $$\varphi_{V}={\left[\left(\frac{1-\varphi_{m}}{\varphi_{m}}\right)\frac{\rho_{p}}{\rho_{w}}+1\right]}^{-1},$$

where: $\rho_{p}$, $\rho_{w}$ are the densities of nanoparticle and water, respectively. $\varphi_{m}$ is the mass concentration for nanofluids. The WO_3_ nanoflakes were utilized as nanomaterial (supplied by US Nanomaterials, Inc., Houston, TX USA) with a thickness of 30 nm, yellow color, and 99.95% purity as provided by the vendor. The scanning electron microscope of tungsten oxide nanopowder is shown in Figure 1. It can be seen that the morphology of the nanomaterial is a flaky shape with a thickness of 25–35 nm, which reveals good agreement with the supplier specifications.

**Figure 2:**
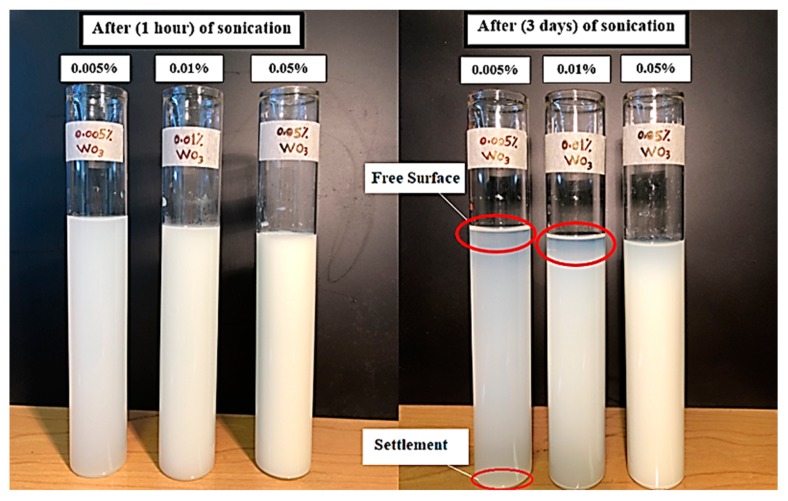
Stability of WO_3_-based water nanofluids with different periods.

One of the most critical stages during the formation of nanofluids is to obtain a stable suspension. However, two physical methods were adopted in this study to disperse the nanopowder inside deionized water. The first method is to mix the nanofluids with a magnetic stirrer for 1 h. Secondly, the ultra-sonication probe (type: Bendelin, SONOPLUS, Berlin, Germany) was used for 45 min to split up the agglomeration of nanoflakes and get a stable suspension. Consequently, we checked the stability of prepared nanofluids with all volume concentrations used in this study. A visualization and time sediment method was adopted to see the stability of the nanofluids after a period of 1 hour and a period of 3 days of the sonication process as shown in Figure 2. The stability was considered quite good to conduct our experiments because our test starts after the sonication process directly.

### 2.2. Pool Boiling Chamber Setup and Procedure

**Figure 3:**
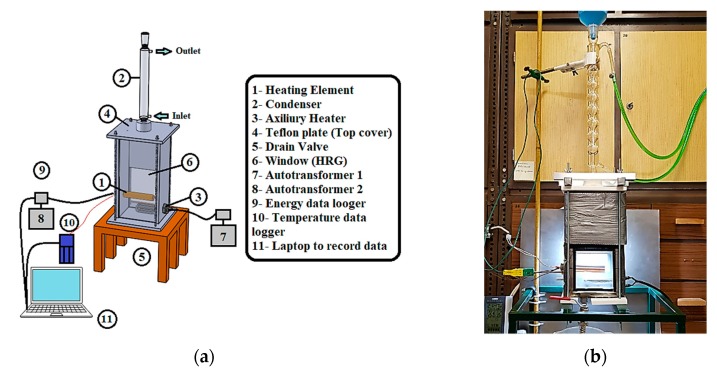
(**a**) Schema of the pool boiling apparatus, (**b**) real picture of the pool boiling apparatus.

The pool boiling setup consists of a heating element (horizontal heated copper tube), boiling chamber, an auxiliary heater to heating the fluid to the saturation temperature, and reflex condenser, as illustrated in Figure 3. The pool boiling chamber used for this study is a stainless steel vessel (dimensions: L = 155 MM, W = 120 mm, H = 310 mm). Fireproof and heat resistant ceramic glass (from Poly M Hungary Ltd., Budapest, Hungary) was utilized to visualize the motion of the bubble during pool boiling experiments from the front side of the chamber. The entire boiling chamber excluding a window with an area of 0.0156 m^2^ was used for visualization purposes covered with two layers of insulation materials. The first layer consisted of solflex, an insulation sheet with a high reflective material on one side (Thermofoam company. OBI store, Budapest, Hungary), the second layer consisted of a 10 mm thermal insulation sheet to minimize heat losses. A cooling condenser (Allihn type) supplied from aalabor.hu was utilized to condense the vapor phase and keep the atmospheric pressure condition as well as the fluid capacity inside the apparatus during the experiments. The main part of this facility is the heating surface, which is a horizontal copper tube with an external diameter of 22 mm and a tube thickness of 1 mm filled with copper sleeve fabricated in our laboratory. The sleeve made from the rigid copper shaft and three grooves were fabricated along with the axial distance with different radial angles and locations. Four K-Type thermocouples were calibrated and used to measure the bulk and surface temperatures. Great efforts were made to fix them in a proper way to measure the surface temperatures without any effect on the surface characteristics of the external tube. Cartridge heater with a power of 1 kW and a diameter of 12.5 mm and a length of 100 mm manufactured by Cartridge heaters, Birmingham, UK, was inserted to the inside diameter of the copper sleeve to supply the heat flux. All facilities above were designed, fabricated, and collected to build up our pool boiling apparatus at the laboratory of the Energy Engineering Department at Budapest University of Technology and Economics, Budapest, Hungary.

**Figure 4:**
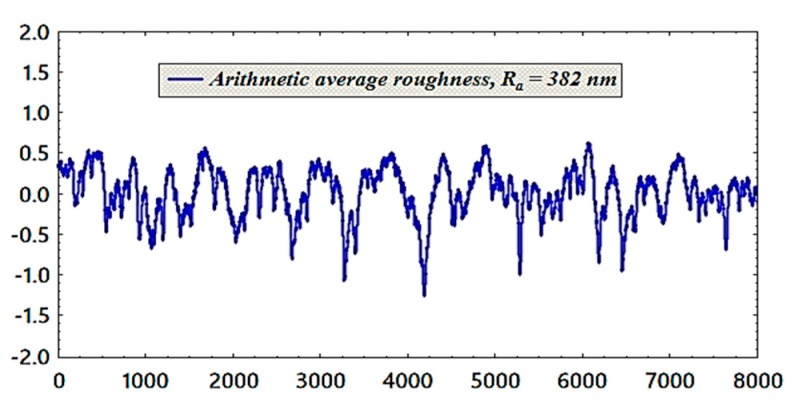
Roughness profile for polished copper tube.

Before starting the tests, the pool boiling chamber was cleaned with a water jet and dried to remove all dirt. Next, the roughness of the heating surface was measured by a surface roughness tester (type: Mitutoyo SJ-400, Mitutoyo, Japan) to obtain the roughness of the polished copper tube before the boiling process. The average of six measurements along the tube with different radial and axial locations were taken to calculate the arithmetic mean roughness parameter (R_a_). The roughness was 0.382 μm, as shown in Figure 4. Afterwards, the working fluid was injected through an injection hole from the top of the chamber. Following this, to heat the liquid to the saturation temperature, the auxiliary heater with a capacity of 400 W was switched on. When the temperature of the bulk fluid reached the saturation temperature, removing any dissolved bubbles inside the chamber, the boiling process was kept for 10 min to remove all those dissolving bubbles. In this research, the boiling points at the atmospheric condition were measured for both water and nanofluids in the same condition. Therefore, the measurements revealed that the water boiling point was higher than the nanofluid boiling point by about 0.2 °C, and this was within the range of the error of the temperature logger. Hence, the boiling point for both deionized water and nanofluid has been assumed to be the same, and this was observed in earlier studies of [19,38]. The next stage was to switch on the cartridge heater with a low heat flux and to keep the auxiliary heater at a specific power of about 20–35 W to maintain the saturation temperature during the initial heat flux stage. The readings of electrical power and temperatures were taken via data acquisition programs after reaching a steady-state condition (about 5 min). The capacity of the working fluids were measured before and after the boiling tests to examine the functionality of the cooling condenser, which is used in this system. The maximum power for cartridge heater used in this experiment was about 1000 W, as mentioned above. Hence, our tests were conducted under the critical heat flux point, which indicates that our scope of this study is to calculate the pool boiling heat transfer coefficient for different working fluids (water and nanofluids). All the measured data for this experiment were taken three times, and the average value was calculated to ensure the repeatability of the results and the average values were used to calculate the applied heat flux and temperatures to find out the PBHTC.

### 2.3. Data Reduction and Uncertainty Analysis

To estimate the pool boiling heat transfer coefficient in the present study, the applied heat flux on the heating element should be calculated by knowing the applied electric power and surface area of the tube, as shown in Equation (2).

(2) $$q^{.}=\frac{V.I}{\pi \;D_{out}.L_{tube}}=\frac{P}{\pi \;D_{out\;}.L_{tube}},$$

where: P is the power in (W), $D_{out\;},\;L_{tube}$ are the outer diameter and length of copper tube in (m), respectively. The surface temperatures were taken from the three thermocouples, which are installed on the outer tube surface. Efforts were made in this experiment to fix the thermocouples in the proper position on the tube surface and to avoid the surface effects on temperature reading. Therefore, the average of the surface temperatures values for top, side, and bottom thermocouples were calculated as shown in Equation (3).

(3) $$T_{s,\;ave}=\frac{T_{\;top}+T_{side}+T_{bottom}}{3},$$

where: $T_{top},\;T_{\;side}$, and $T_{\;bottom}$ are the surface temperatures of the top, side, and bottom thermocouples locations, respectively. The pool boiling heat transfer coefficient was calculated from the above-mentioned physical quantities as shown in Equation (4).

(4) $$\mathrm{PBHTC}=\frac{q^{.}}{\Delta T_{sup}}=\frac{q^{.}}{\left(T_{ave}-T_{sat}\right)},$$

where: $T_{ave},\;T_{sat}$ are the average surface temperature and saturation temperature, respectively. $\Delta T_{sup}$ is the superheat temperature, which is the difference between surface and saturation temperatures. The uncertainty analysis for multiple measured variables for this experiment were calculated according to [39] as shown in Equation (5).

(5) $$\Delta U=\sqrt{{\left(\frac{\partial U}{\partial X_{1}}\Delta X_{1}\right)}^{2}+{\left(\frac{\partial U}{\partial X_{2}}\Delta X_{2}\right)}^{2}+{\left(\frac{\partial U}{\partial X_{3}}\Delta X_{3}\right)}^{2}},$$

where: $X_{1},\;X_{2}$, and $X_{3}$ are the variables. $\Delta X_{1}$, $\Delta X_{2}$, and $\Delta X_{3}$ are the uncertainties given by instrument data-sheets. Therefore, The relative uncertainties for the applied heat flux and pool boiling heat transfer coefficient were found to be about ±1.1% and ±5.5% respectively.

## 3. Results and Discussion 

In this section, the pool boiling heat transfer performance for deionized water and tungsten oxide nanoflake-based deionized water nanofluids with dilute volume concentration from a typical horizontal heated copper tube at atmospheric pressure condition was presented.

### 3.1. Validation of The Experimental Results

**Figure 5:**
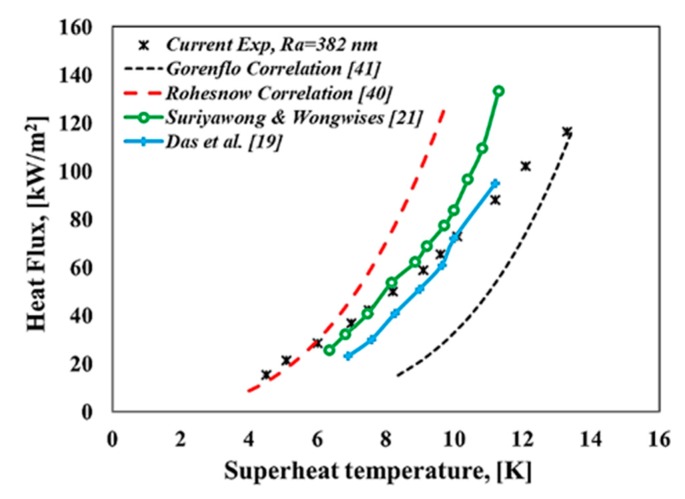
Boiling curves for present experimental research and literature studies.

**Figure 6:**
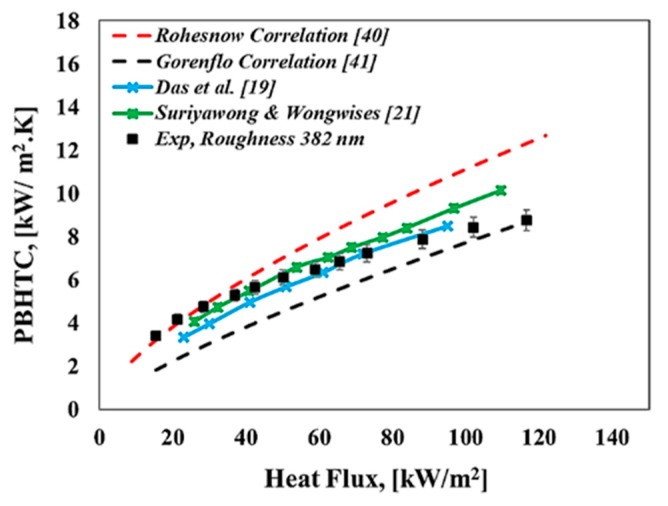
Pool boiling heat transfer coefficient (PBHTC) against heat flux for current research and literature studies.

Prior to discussing our obtained results, the pool boiling apparatus and procedure should be validated to ensure the accuracy of the present results. The results of pool boiling curves and the pool boiling heat transfer coefficient for deionized water were compared to well-known correlations in literature, such as the so-called Rohesnow correlation [40], Gorenflo Correlation [41], Equations (6)–(10), and the experimental studies reported in the literature, such as Das et al. [19], Suriyawong and Wongwises [21] with horizontal heated tubes as heating surfaces. The reason for using deionized water for the validation of our obtained results determined that these type of fluids were well known and had high accurate thermophysical properties in literature [42,43]. Therefore, our experimental results for deionized water validated with the above-mentioned correlations and experimental studies as can be seen from Figure 5 and Figure 6 where the experimental results compared reasonably well with predicted correlations as well as experimental studies for pool boiling curves. Moreover, considering the importance of repeatability, experiments were conducted three times with similar conditions. Results demonstrated that the main pool boiling curves remained unchanged during the repeating procedure.

(6) q.=μlhlv[σg(ρw−ρv)]−0.5(1Csf)10.333Prl10.333[Cp,lΔTsuphlv]10.333,

where: $\mu_{l}$, $\rho_{l}$, and $C_{p,l}$ are the viscosity, density, and specific heat for liquid. $h_{lv}$, σ, and $\rho_{v}$ are the latent heat of vaporization, the surface tension of the liquid, and density of the vapor, respectively. $C_{sf}$ is a constant that depends on surface characteristics, and in this study considered to be 0.0128 for polished copper tube as suggested by [44] and used by [29].

(7) $$\mathrm{PBHTC}={\;\mathrm{PBHTC}}_{0}\times F_{p}\times F_{q}\times F_{SR},$$

(8) $$F_{p}=1.73pr^{0.27}+\left(6.1+\frac{0.68}{1-Pr}\right)\times pr^{2},$$

(9) $$F_{q}={\left(\frac{q^{.}}{q_{0}^{.}}\right)}^{n},\;n=0.9-0.3Pr^{0.15},$$

(10) $$F_{SR}={\left(\frac{Ra}{Ra_{0}}\right)}^{0.133},$$

where: pr, $q^{.},$ and Ra are the reduced pressure ratio $\left(pr=p/p_{c}\right)$, heat flux (kW/m^2^), and average surface roughness for heating surface (μm) respectively. The reference conditions for pure water used in this correlation are ${\mathrm{PBHTC}}_{0}=5600\;\left(\mathrm{kW}/m^{2}K\right)$, $q_{0}^{.}=20\;(\mathrm{kW}/m^{2})$, and ${\mathrm{Ra}}_{0}=0.4\;\left(\mu m\right)$.

### 3.2. Results of Pool Boiling Heat Transfer for WO_3_-Based Water Nanofluids

**Figure 7:**
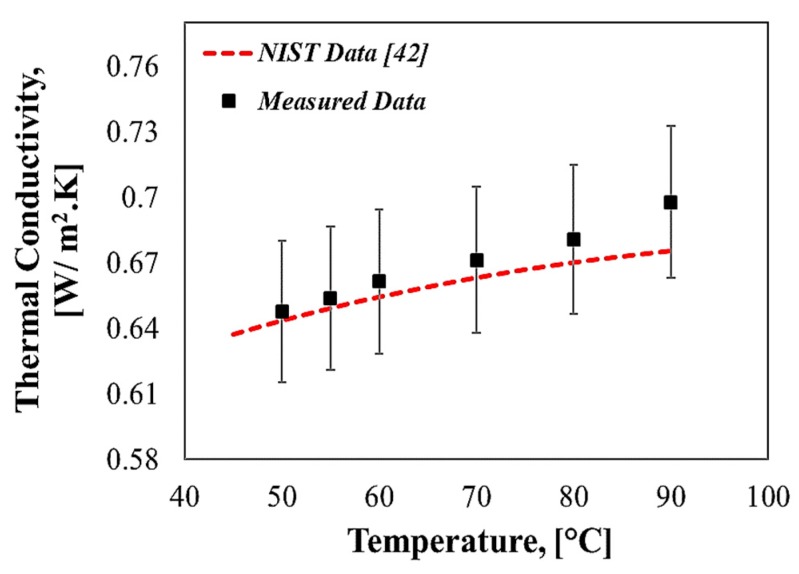
The validation of thermal conductivity results for deionized water with National Institute for Standard and Technology (NIST) data [42].

**Figure 8:**
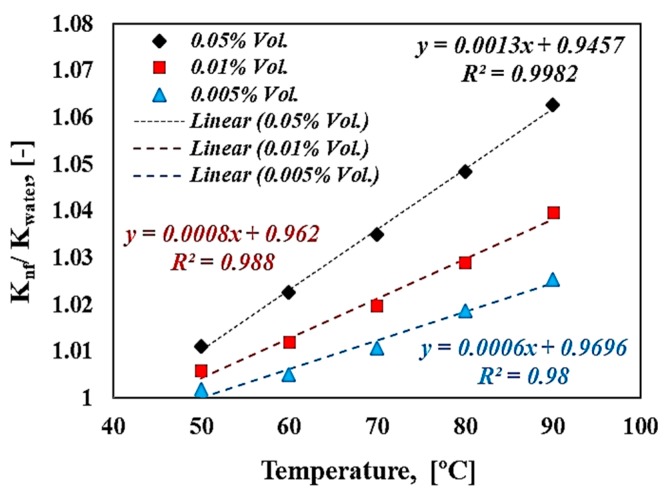
Thermal conductivity ratio at different volume concentrations and temperatures.

It is widely reported in the literature that the boiling heat transfer performance of nanofluids could be affected by the enhancement of the bulk properties, such as the thermal conductivity, surface tension, wettability, viscosity, and latent heat of vaporization [38,45,46,47]. However, in the present study, the effect of bulk fluid properties was assessed, such as thermal conductivity on pool boiling of tungsten oxide-based water nanofluid. Thermal conductivity of nanofluids were measured by using the transient plane heat source method (SKZ1061C thermal conductivity tester, SKZ Industrial Co., Jinan, China) at different temperatures (50–90 °C) and volume concentrations (0.005–0.055 Vol.). The results obtained for deionized water were also used as reference to compare them with the measured data for nanofluids. The validation of the obtained results for a temperature range (50–90 °C) after calibration showed a high accuracy behavior with the thermal conductivity data for deionized water of the National Institute for Standard and Technology (NIST), as presented in Figure 7. It was revealed that the present measurements for deionized water were in good agreement of well-known water thermal conductivity data presented in the literature [42], and the maximum deviation was found to be (3.3%) for higher temperature, and this fell within the range of the sensor accuracy (±5%). Figure 8 shows the thermal conductivity ratio of WO_3_ nanofluids relative to water at different concentrations and temperatures. It can be seen from Figure 8 that the higher enhancement percentage for thermal conductivity was about (6.3%) for nanofluids with a volume concentration of 0.05% Vol. and a temperature of 90 °C. This enhancement was expected because the solid material has a thermal conductivity higher than the thermal conductivity of deionized water and also the Brownian motion of nanoparticle increased with the increase of temperature due to the high kinetic energy. 

**Figure 9:**
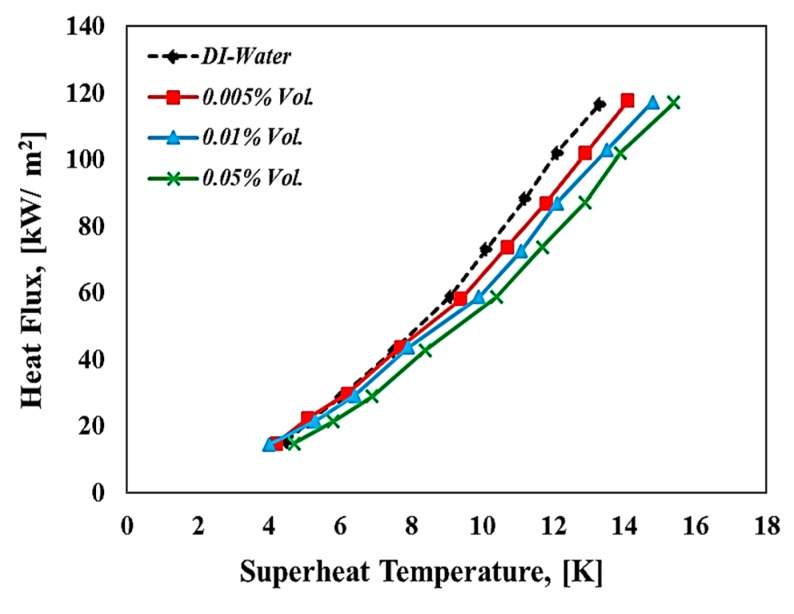
Boiling curves for water and WO_3_-based water nanofluids at different volume concentrations.

Figure 9 illustrates the heat flux against the superheat temperature ($\Delta T_{sup}$), which is called the pool boiling curve for both deionized water and WO_3_-based deionized water nanofluids. The maximum applied heat flux used for all tests in this research did not exceed 130 kW/m^2^, and this means all measurements were done under the critical heat flux value due to the power limitation of the cartridge heater used in this study. It can be seen from Figure 9 that for all working fluids with an increasing applied heat flux, the superheat temperature was also increased with relatively small temperature difference, and this is due to the nucleate pool boiling regime represented by the latent heat of vaporization. For the smallest volumetric concentration (i.e., 0.005% and 0.01% Vol.), especially at the low heat flux region used in this study, the boiling curve is obviously shifted leftward compared to the deionized water pool boiling curve. However, an increase in volume concentration of nanofluids (i.e., 0.05% Vol.) considerably shifted the boiling curve shifted to the right side, and this change was notable especially in high heat flux regions above 50 kW/m^2^. The higher superheat temperatures at the same applied heat flux mean the pool boiling performance degraded and this could be attributed to the deposition of the nanoflakes on the heating surface during the boiling process. At high heat flux, the formation of the bubble increased due to the increase of the nucleation site density. Hence, the deposition of the tungsten oxide nanoflakes increases due to the microlayer evaporation mechanism which occurs during the formation of bubbles.

**Figure 10:**
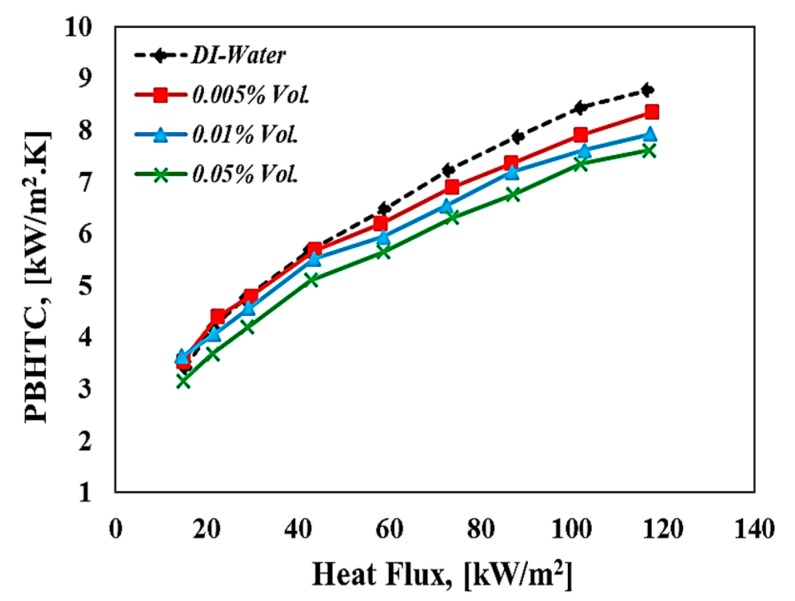
Pool boiling heat transfer coefficient against heat flux of WO_3_-based water nanofluids at different volume fractions.

**Figure 11:**
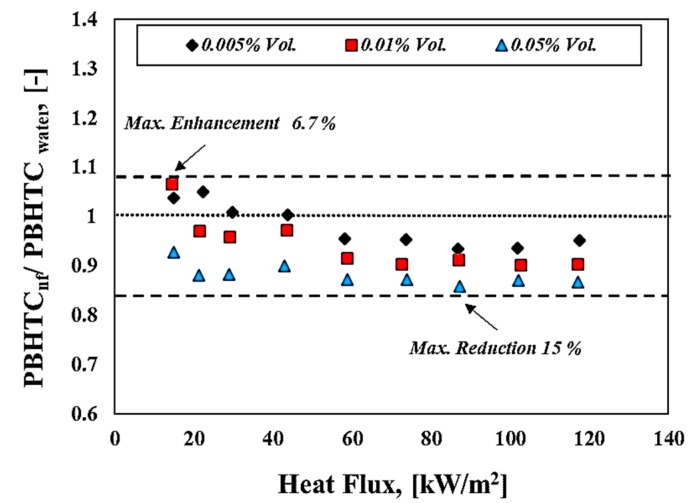
Pool boiling heat transfer coefficient enhancement ratio against heat flux of WO_3_-based water nanofluids at different volume fractions.

Figure 10 shows the relationship between the pool boiling heat transfer coefficient and the applied heat flux for both deionized water and nanofluids. It can be seen that the pool boiling heat transfer coefficient for volume concentration (i.e., 0.005% and 0.01% Vol.) slightly increases for low heat flux region, and this could be attributed to the bulk effect for tungsten oxide nanofluids that are represented by thermal conductivity enhancement. Meanwhile, the increasing volume concentration led to a reduction in pool boiling heat transfer coefficient compared to water, and this reduction attributed to the deposition of nanoflakes on the surface which, in turn, built a resistance thermal layer to transfer the heat from the heating surface toward the working fluid. This trend was also reported by some researchers [19,22,32]. Moreover, as mentioned above, the deposition of those nanoflakes decreased the boiling behavior by deactivating the nucleation sites and then changing the bubble formation and their dynamic behavior during the boiling process. Figure 11 presents the pool boiling heat transfer coefficient ratio (${\mathrm{PBHTC}}_{\mathrm{nf}}/{\mathrm{PBHTC}}_{\mathrm{water}}$) of nanofluid relative to pure water at various volume concentrations. It can be seen from Figure 11 that there is an enhancement in this ratio up to (6.7%) for dilute concentrations (i.e., 0.01% Vol.) of WO_3_ nanoflake-based water nanofluid at low heat flux. In addition, it can be observed that the behavior of pool boiling heat transfer coefficient of nanofluids at low heat flux (free convection regime) was better than in the case of water, especially for volume concentrations 0.005% and 0.01% Vol. where the bulk effect dominates in this region. For high heat fluxes (>40 kW/m^2^), which means at the nucleate pool boiling regime, there is a considerable decrease in the pool boiling heat transfer coefficient of nanofluids compared to deionized water at all volume concentrations used in this study. The maximum reduction in pool boiling heat transfer coefficient ratio was about 15% at a volume fraction of 0.05% Vol., relative to pure water case. It was reported in the literature that pool boiling heat transfer performance could be affected by the modification of the heating surface (i.e., surface roughness, wettability, and capillary wicking forces) as well as the bulk effect associated to thermal properties (i.e., thermal conductivity, viscosity, and surface tension). However, a surface modification that resulted from the deposition of the nanopowder during the boiling process has a significant role in causing degradation or improvements for the boiling heat transfer performance of nanofluids. Many parameters have a direct influence on the pool boiling heat transfer coefficient, such as the heating surface materials, pressure system, nanomaterials type, shapes, size, and concentrations [22,48,49,50]. The size and shape of nanomaterials are considered to be important parameters that might affect the surface characteristics during the boiling process. Those parameters have a direct interlink with surface roughness utilizing the surface particle interaction parameter that is introduced and discussed by [37].

**Figure 12:**
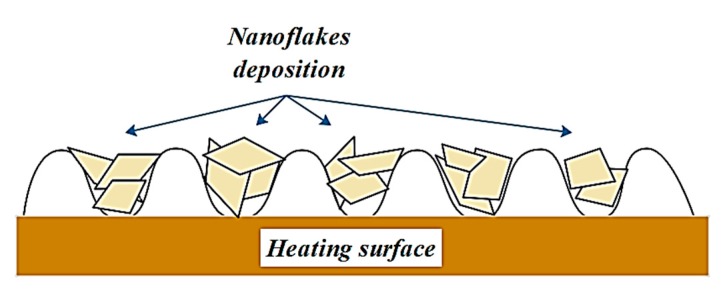
Schema of tungsten oxide WO_3_ nanoflakes deposition on the heating surface.

**Figure 13:**
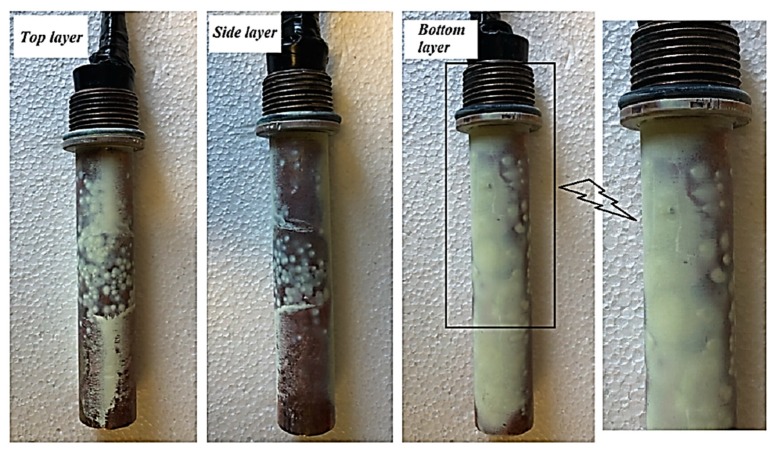
Deposition nanolayer of WO_3_ nanoflakes on different radial and axial locations at 0.05% Vol. nanofluid concentration.

Figure 12 and Figure 13 show, schematically and photographically, the deposition of WO_3_ nanoflakes on the heating surface during the pool boiling heat transfer process. It can be observed from Figure 12 that the deposition of those nanoflakes on the heating surface could cause the deactivation of the nucleation site by lying those nanoflakes on the microcavities of the surface, which in turn resulted in a reduction of bubble formation and distribution, especially at the nucleate boiling region. On the other hand, the deposition of those nanoflakes creates a porous nanolayer on the heating surface which could work as a thermal resistance layer that hinders the heat transfer from the surface toward the bulk fluid. Thus, for better understanding regarding this hypothesis, photos were taken to capture the deposition of that nanopowder on the horizontal heat tube that was used as a heating element in the present research. Figure 13 illustrates the deposition of the WO_3_ nanoflakes after the pool boiling test with a volume concentration of 0.05% Vol., which was considered a higher concentration in this study. The built nanolayer from three directions of the horizontal heated tube was detected and it can be seen that the yellow layer on the heated tube dominated the action on the bottom side, and this could be attributed to the nature of the bubble formation and sliding from the horizontal tube, where the bubbles start to form from the bottom side growing and then sliding on both sides of the tube, and finally, at a certain point, elapse from the tube towards to the bulk fluid as studied by the research of [51]. According to findings of [36], the deposition of nanopowder led to creating the nanoporous layer on the heating surface that makes the heating surface hydrophilic, which in turn increases the wettability and decreases the contact angle. As mentioned above, the porous structure may enhance or deteriorate the pool boiling process depending on the surface particle interaction parameter. In the present study, it was hypothesized that the deposition of the nanoflakes changes the surface characteristics (decrease the roughness of the surface), as shown in Figure 12, and this was proven as shown in Figure 13. It can be stated that the deposition of nanoflakes on heating surfaces reduced the number of nucleation sites by decreasing the roughness of the surface. On the other hand, this deposition with such shape and size of nanopowder creates an extra thermal resistance layer. Hence, these two observations were the most important issues to reduce the heat transfer performance during the pool boiling of tungsten oxide-based water nanofluid.

## 4. Conclusions

In this experimental research, the pool boiling heat transfer performance of deionized water and tungsten oxide WO_3_ based deionized water nanofluids with various dilute volumetric concentrations at atmospheric pressure was examined. The pool boiling heat transfer coefficient enhancement ratio of nanofluids relative to deionized water was introduced to see the effect of nanofluid concentration and applied heat flux on pool boiling heat transfer behavior. Results found that the pool boiling heat transfer coefficient ratio was enhanced by about 6.7% at volume concentrations of 0.005% and 0.01% Vol. in the low heat flux region, while there was a reduction in this ratio by about 15% at a volume concentration of 0.05% Vol. in the high heat flux region. The enhancement ratio could be attributed to the bulk effect represented by the enhancement of the thermal conductivity property. Whereas, the reduction ratio could be a result of the nanoflakes deposition during the pool boiling process, which in turn filled the microcavities of the surface and reduced the nucleation sites density, as well as this layer, and restricted the heat transfer from the surface to the working fluid due to the low thermal conductivity of the created nanoporous layer.

## Nomenclatures

PBHTC	pool boiling heat transfer coefficient, kW/m^2^.K
EG	ethylene glycol
*CNTs*	carbon nanotubes
$\varphi_{V}$	volume concentration, %
$\varphi_{m}$	mass concentration, %
$\rho_{p}$	the density of nanopowder, kg/m^3^
$\rho_{w}$	the density of water, kg/m^3^
$\rho_{v}$	the density of vapor, kg/m^3^
L	length of the boiling chamber, m
W	width of the boiling chamber, m
H	the height of the boiling chamber, m
$R_{a}$	arithmetical mean roughness value, μm
$q^{.}$	applied heat flux, kW/m^2^
P	electrical power, W
$D_{out\;}$	the outer diameter of the copper tube, m
$L_{tube}$	length of copper tube, m
$T_{top}$	the temperature at the top surface, °C
$T_{side}$	the temperature at the side surface, °C
$T_{bottom}$	the temperature at the bottom surface, °C
$T_{s,ave}$	average surface temperature, °C
$\Delta T_{sup}$	superheat temperature, K
$T_{sat}$	saturation temperature, °C
$\mu_{l}$	the viscosity of the liquid, Pa.s
$h_{lv}$	latent heat of vaporization, kJ/kg
σ	surface tension, N/m
$C_{p,l}$	specific heat of the liquid, kJ/kg. K
$C_{sf}$	liquid–surface interaction coefficient
$Pr_{l}$	liquid Prandtl number
pr	reduced pressure ratio
$F_{p}$, $F_{q}$, $F_{SR}$	constants in Gorenflo correlation.
**Subscripts**	
*V*	volume
*m*	mass
*p*	powder
*w*	water
*v*	vapor
*l*	liquid
*out*	outer
*s*	surface
*sup*	superheat
*sat*	saturation
*nf*	nanofluid
*0*	reference condition
